# Complete Genome Sequence of a Hyperthermophilic Archaeon, *Thermosphaera* sp. Strain 3507, Isolated from a Chilean Hot Spring

**DOI:** 10.1128/MRA.01262-20

**Published:** 2020-12-10

**Authors:** Kseniya S. Zayulina, Alexander G. Elcheninov, Stepan V. Toshchakov, Ilya V. Kublanov

**Affiliations:** a Winogradsky Institute of Microbiology, Research Center of Biotechnology RAS, Moscow, Russia; b National Research Center Kurchatov Institute, Moscow, Russia; Portland State University

## Abstract

A complete genome sequence of a hyperthermophilic archaeon, *Thermosphaera* sp. strain 3507, which was isolated from a Chilean hot spring, is presented. The genome is 1,305,106 bp with a G+C content of 47.6%. Twenty-seven carbohydrate-active enzyme genes were identified, which is in accordance with the ability of the strain to grow on various polysaccharides.

## ANNOUNCEMENT

The genus *Thermosphaera* is affiliated with the *Desulfurococcaceae* family (1) of the *Crenarchaeota* phylum and is currently represented by a single species, Thermosphaera aggregans M11TL^T^ (2). Strain 3507 was isolated from a sample of mud and water collected from a hot spring (temperature, 83°C [pH 6.3]; 34°57.518′S, 70°26.331′W) located in the Termas del Flaco area within the Tinguiririca volcano thermal zone in Chile (3). Strain 3507 was isolated by a serial dilution technique from a binary enrichment culture obtained by incubation of the sample for 7 days at 85°C in anaerobic Pfennig medium (4) with twice-reduced salt concentrations and supplemented with lichenan (1g liter^−1^) (pH 6.5).

For genomic sequencing, the strain was cultured for 3 days at 85°C at pH 6.5 in medium (4) supplemented with lichenan (1g liter^−1^). Genomic DNA isolation was performed using a Genomic-tip 20/G (Qiagen), according to the manufacturer’s instructions. Approximately 100 ng of isolated DNA was used for fragment library preparation with the Nextera DNA Flex library preparation kit (Illumina), according to the manufacturer’s protocol. The library was sequenced with the Illumina MiSeq system, using a 2 × 150-bp sequencing kit; 1,159,189 read pairs were obtained from a sequencing run. Reads were subjected to quality filtering and trimming with the Trim Reads tool of CLC Genomics Workbench v20.0.4 (Qiagen), using zero maximum ambiguities and 0.01 error probability. Trimming of sequencing adapters and merging of overlapping read pairs were performed with the SeqPrep tool (https://github.com/jstjohn/SeqPrep). A total of 710,804 read pairs and 396,658 merged reads were used for *de novo* assembly with the SPAdes v3.14.1 assembler in the “--isolate” mode (5). One contig of 1,306,603-bp length was obtained. Circularization was performed by broken read pair analysis with CLC Genomics Workbench v20.0.4 and the CLC Genome Finishing Module (Qiagen). Finally, one circular 1,305,106-bp chromosome was obtained. The start of the chromosome was set to the origin of replication predicted by the OriFinder 2 tool (6). Genome annotation was performed with PGAP (7). The average amino acid identity (AAI) and average nucleotide identity (ANI) were calculated using the AAI.rb script (8) and the pyani module v0.2.8 (9), respectively. Carbohydrate-active enzymes (CAZymes) were identified with dbCAN2 (10). Amino acid biosynthetic pathways were predicted by GapMind (11).

The final assembly of the strain 3507 genome comprises a single circular chromosome with a length of 1,305,106 bp and a G+C content of 47.6%. In total, 1,458 genes were predicted, including 1,399 protein-coding genes, 50 RNA genes (3 rRNA genes, 45 tRNA genes, and 2 noncoding RNA genes), and 9 pseudogenes. A BLAST search revealed 99.67% 16S rRNA sequence identity with Thermosphaera aggregans M11TL^T^; however, pairwise AAI and ANI values were 86.5% and 83.2%, respectively, which are below the species-level thresholds. The capability of polysaccharide utilization by the strain is in accordance with the presence of the CAZyme genes in its genome. The CAZymes were represented by 10 glycosidases (glycoside hydrolase 1 [GH1], GH13, GH57, and GH122 families) and 17 glycosyltransferases; 13 of them are predicted to be secreted. The genome analysis revealed probable arginine, histidine, lysine, methionine, proline, serine, branched-chain amino acid, and aromatic amino acid auxotrophy.

### Data availability.

The whole-genome sequence was deposited in DDBJ/ENA/GenBank under the accession number CP063144.1. The BioProject, BioSample, and SRA accession numbers are PRJNA668939, SAMN16428067, and SRR12969965, respectively.
